# Tea leaf disease detection and identification based on YOLOv7 (YOLO-T)

**DOI:** 10.1038/s41598-023-33270-4

**Published:** 2023-04-13

**Authors:** Md. Janibul Alam Soeb, Md. Fahad Jubayer, Tahmina Akanjee Tarin, Muhammad Rashed Al Mamun, Fahim Mahafuz Ruhad, Aney Parven, Nabisab Mujawar Mubarak, Soni Lanka Karri, Islam Md. Meftaul

**Affiliations:** 1 Department of Farm Power and Machinery, Sylhet Agricultural University, Sylhet, 3100 Bangladesh; 2 Department of Food Engineering and Technology, Sylhet Agricultural University, Sylhet, 3100 Bangladesh; 3 Department of Agricultural Construction and Environmental Engineering, Sylhet Agricultural University, Sylhet, 3100 Bangladesh; 4 Global Centre for Environmental Remediation (GCER), College of Engineering, Science and Environment, The University of Newcastle, Callaghan, NSW 2308 Australia; 5 Department of Agricultural Chemistry, Sher-e-Bangla Agricultural University, Dhaka, 1207 Bangladesh; 6 Petroleum and Chemical Engineering, Faculty of Engineering, Universiti Teknologi Brunei, Bandar Seri Begawan, BE1410 Brunei Darussalam; 7 Faculty of Integrated Technologies, Universiti Brunei Darussalam, Bandar Seri Begawan, BE1410 Brunei Darussalam

**Keywords:** Plant sciences, Environmental sciences, Environmental social sciences, Planetary science

## Abstract

A reliable and accurate diagnosis and identification system is required to prevent and manage tea leaf diseases. Tea leaf diseases are detected manually, increasing time and affecting yield quality and productivity. This study aims to present an artificial intelligence-based solution to the problem of tea leaf disease detection by training the fastest single-stage object detection model, YOLOv7, on the diseased tea leaf dataset collected from four prominent tea gardens in Bangladesh. 4000 digital images of five types of leaf diseases are collected from these tea gardens, generating a manually annotated, data-augmented leaf disease image dataset. This study incorporates data augmentation approaches to solve the issue of insufficient sample sizes. The detection and identification results for the YOLOv7 approach are validated by prominent statistical metrics like detection accuracy, precision, recall, mAP value, and F1-score, which resulted in 97.3%, 96.7%, 96.4%, 98.2%, and 0.965, respectively. Experimental results demonstrate that YOLOv7 for tea leaf diseases in natural scene images is superior to existing target detection and identification networks, including CNN, Deep CNN, DNN, AX-Retina Net, improved DCNN, YOLOv5, and Multi-objective image segmentation. Hence, this study is expected to minimize the workload of entomologists and aid in the rapid identification and detection of tea leaf diseases, thus minimizing economic losses.

## Introduction

Tea is one of the world's most popular functional beverages due to its pleasant flavor, exquisite taste, and biological benefits. It contains several active phyto-constituents that have significant benefits for human health. The most intriguing fact is that it has become the most consumed beverage (next to water)^1^. Tea plays an important role in bringing families and friends closer together across the world^2^. By 2025, global tea consumption is anticipated to reach 7.4 M MT, up from approximately 7.3 M MT in 2020^3^.

The demand for tea production will increase in the coming days. In contrast, the production of tea is declining due to weather conditions and climate change. Besides these global phenomena, various diseases and pests badly affect tea production and quality. Diseases frequently afflict tea plants during their development and growth. Over one hundred prevalent diseases are identified worldwide damaging the tea leaves^4^. Tea is amongst the superior agro-industrial and export-oriented crops of Bangladesh. It is regularly consumed by most of the country's people, and its flavor is well-liked within and beyond its country of origin^5^. Bangladesh has 162 tea gardens divided into two main tea-growing regions: Sylhet in the northeast and Chittagong in the south^5^. Bangladesh's enormous tea production has undoubtedly helped its GDP while positioning it as the world's leading tea exporter.

The early and accurate diagnosis of plant diseases and pests significantly prevents agricultural production losses. If tea leaf diseases are accurately and rapidly identified, they can be prevented and managed more efficiently^6^. In recent times, tea leaf disease diagnosis has been performed manually. Because the bulk of tea plants grow in tough hilly terrain, it is time-consuming and expensive for professionals to visit tea gardens for diagnosis. When farmers rely on their personal experiences to differentiate between different forms of tea diseases, the outcomes are highly subjective^7^. The accuracy of such projections is low, and identifying diseased leaves requires substantial work. Therefore, a framework should allow for more precise and reliable disease diagnosis^6^.

With the advancement of computing technology, machine learning and image processing can automatically detect and identify plant diseases, playing a significant role in the automatic diagnosis of plant diseases^8,9^. Researchers have applied image processing and machine learning to identify and categorize plant diseases. Castelao Tetila et al. applied six traditional machine-learning approaches to detect infected soybean leaves captured by an Unmanned Aerial Vehicle (UAV) from various heights. The impact of color and texture features was validated based on the recognition rate^10^. Maniyath et al.^11^, suggested a classification architecture based on machine learning to detect plant diseases. In another recent study, Ferentinos^12^ used simple leaf images of healthy and infected plants and constructed convolutional neural network models for plant disease identification and diagnosis using deep learning. Fuentes et al.^13^ employed "deep learning meta-architectures" to identify diseases and pests on tomato plants by utilizing a camera to capture images with varying resolutions. As a result of fruitful investigations, the approaches continued to detect nine distinct types of tomato plant diseases and pests. Tiwari et al.^14^ introduced a dense convolutional neural network strategy for detecting and classifying plant diseases from leaf pictures acquired at different resolutions. This deep neural network addressed many inter-class and intra-class variances in images under complicated circumstances. Several additional studies have utilized deep learning and image-processing techniques to identify tea leaf diseases. Hossain et al.^15^ discovered an image processing method capable of analyzing 11 features of tea leaf diseases and utilized a support vector machine classifier to identify and classify the 2 most common tea leaf diseases, namely brown blight disease and algal leaf disease. Sun et al.^16^ improved the extraction of tea leaf disease saliency maps from complicated settings by combining simple linear iterative cluster (SLIC) and support vector machine (SVM). Hu et al.^17^ developed a model for analyzing the severity of tea leaf blight in natural scene photos. The initial disease severity (IDS) index was calculated by segmenting disease spot locations from tea leaf blight leaf images using the SVM classifier. Additionally, various researchers have used notable architectures, such as AlexNet^18^, VGGNet^19^, GoogLeNet^20^, InceptionV3^21^, ResNet^22^, and DenseNet^23^, for plant disease identification.

While the abovementioned techniques have proven effective in treating crop or plant diseases, they are limited to diagnosing or classifying crop disease images. As mentioned earlier, deep neural networks are ineffective in detecting and recognizing tea leaf diseases in images of natural scenes. This is because natural scene images of tea leaves contain complex backgrounds, dense leaves, and large-scale alterations. One-stage algorithms performed well compared to the other deep learning models^24^. Recently, image detection networks based on deep learning have been separated into two-stage and one-stage networks^24^. The first is the R–CNN (region-based convolutional neural network) family of algorithms, which are geared for regional proposals and comprise representative networks such as R–CNN, Fast R–CNN, Faster R–CNN, Mask R–CNN, etc. Another category is one-stage algorithms and their representative networks, such as the YOLO (you only look once) series^25^.

YOLO is an object detection algorithm that has gained popularity in computer vision. YOLO is a real-time object detection algorithm that processes an image in a single forward pass through a neural network. Unlike traditional object detection algorithms involving multiple processing stages, YOLO performs object recognition and bounding box regression in a single step^24^. This makes it fast and efficient, with the ability to process up to 60 frames per sec. YOLO works by dividing an image into a grid of cells and predicting bounding boxes for each cell. For each bounding box, YOLO predicts the class probability (i.e., the probability that the bounding box contains a particular object) and the confidence score (i.e., the probability that the bounding box contains an object). YOLO also predicts the bounding box coordinates relative to cell^26^.

To improve the accuracy of the predictions, YOLO uses a technique called anchor boxes, which are predefined boxes of different sizes and aspect ratios. Each anchor box is associated with a particular cell and is used to predict the size and shape of the object within that cell. Using anchor boxes, YOLO can handle objects of different sizes and shapes. One of the main strengths of YOLO is its speed. YOLO can process images in real-time, which makes it suitable for applications such as autonomous vehicles, surveillance systems, and robotics^26^. YOLO is also efficient, as it only needs to process an image once, unlike traditional object detection algorithms that require multiple passes through the network. Another strength of YOLO is its ability to detect multiple objects in an image. Because YOLO predicts bounding boxes for each cell, it can detect multiple objects in different image parts. This makes YOLO ideal for pedestrian detection and traffic sign recognition^26^.

YOLOv7 is the new advanced detector in the YOLO family. This network uses trainable bag-of-freebies, allowing real-time detectors to improve precision dramatically without increasing inference costs. It integrates extend and compound scaling, allowing the target detector to effectively reduce the number of parameters and calculations, resulting in a substantial acceleration of the detection rate^27^. YOLOv7 exceeds typical object detectors in precision and speed of 5 FPS (frames per sec) to 160 FPS. It also gives a set of ready-to-use freebies and makes it simple to fine-tune detection models. YOLOv7's configuration file makes it straightforward to add additional modules and generate new models^28^. The study offers E-ELAN, which employs expand, shuffle, and merge cardinality to accomplish the capacity to constantly improve the network's learning ability without breaking the original gradient path^29^.

The previous version of the YOLO family (YOLOv5) has been applied effectively in various domains, including fruit identification by harvesting robots^30,31^, vehicle and ship detection^32,33^, poisonous mushroom selection, and face detection^34^. Jubayer et al.^35^ used YOLOv5 for mold detection and demonstrated precision, recall, F1, and AP of 98.1%, 100%, 99.5%, and 99.6%, respectively.

The upgraded YOLO (YOLOv7) variant has caught many machine learning and data modelling scientists. Several researchers have employed for various object-detecting phenomena, such as video object tracking^29^, object detection for Hemp duck count estimation^36^, object detection of maritime UAV images^37^, ship detection from satellite images^38^, defect detection in different materials^39–41^, vehicle tracking^42^, as well as in healthcare^43,44^. Gallo et al.^45^ applied the YOLOv7 model on a dataset of Chicory plants to identify weeds. Similarly, YOLOv7 architecture was used to detect fruits in orchards, making it easier for harvesting robots to locate and collect fruits^46,47^.

The YOLO family has been extensively utilized to identify leaf diseases and insect pests in crops, encouraging us to consider YOLOv7 as a baseline model. The YOLOv7 algorithm is not yet used for identifying tea leaf diseases. For the continuation of this research, the following knowledge gaps are considered:

1. Limited labelled data is available for detecting tea leaf disease, training, and testing any model.
2. There is a lack of established evaluation metrics or benchmarks specific to tea leaf disease detection, making it difficult to compare the performance of YOLOv7 models to other methods
3. While there have been few studies on the application of artificial intelligence for tea diseases, none have been undertaken in Bangladesh. It is crucial to look into the potential benefits and efficacy of utilizing AI to identify and detect tea leaf diseases in Bangladesh.

The present study was designed to identify and detect tea leaf diseases using images captured in the natural environment of numerous tea estates in the Sylhet region of Bangladesh. This paper uses diseased tea leaves as the research object, collects five types of frequent flaw images to produce a tea leaves flaw dataset, and applies the high detection speed and accuracy of the YOLOv7 algorithm to the field of object detection. This research intends to develop an automated method for detecting, identifying, and classifying tea plant diseases, increasing the precision of disease detection, saving farmers time, and benefiting their livelihoods. According to our knowledge, this is the first time YOLOv7 with the attention model has been used as the fundamental architecture for detecting diseased leaves in tea plants.

The main contributions of our work are as follows:

1. We present an improved YOLOv7 object detection model, YOLO-T, for the automatic detection, identification, and resolution of the problem of automatic detection accuracy of tea leaf diseases in images of natural scenes.
2. The performance of the developed YOLO-T was evaluated to the previous version of YOLO (YOLOv5). The present study and previous plant disease detection algorithms are also compared.
3. We create and present an original dataset of images of diseased tea leaves obtained from the prominent tea gardens of Sylhet, Bangladesh. This brand-new dataset might be used for training and testing the YOLOv7 model and by other researchers working on comparable problems.
4. The data augmentation technique is used to increase the number of training images to address the issue of insufficient samples and enhance the network's detection and identification effect.
5. This study's methodology provides a foundation for the automatic prevention and management of tea leaf diseases and facilitates the sensible application of pesticides utilizing drone technology.

## Materials and methods

### Study area

**Figure 1:**
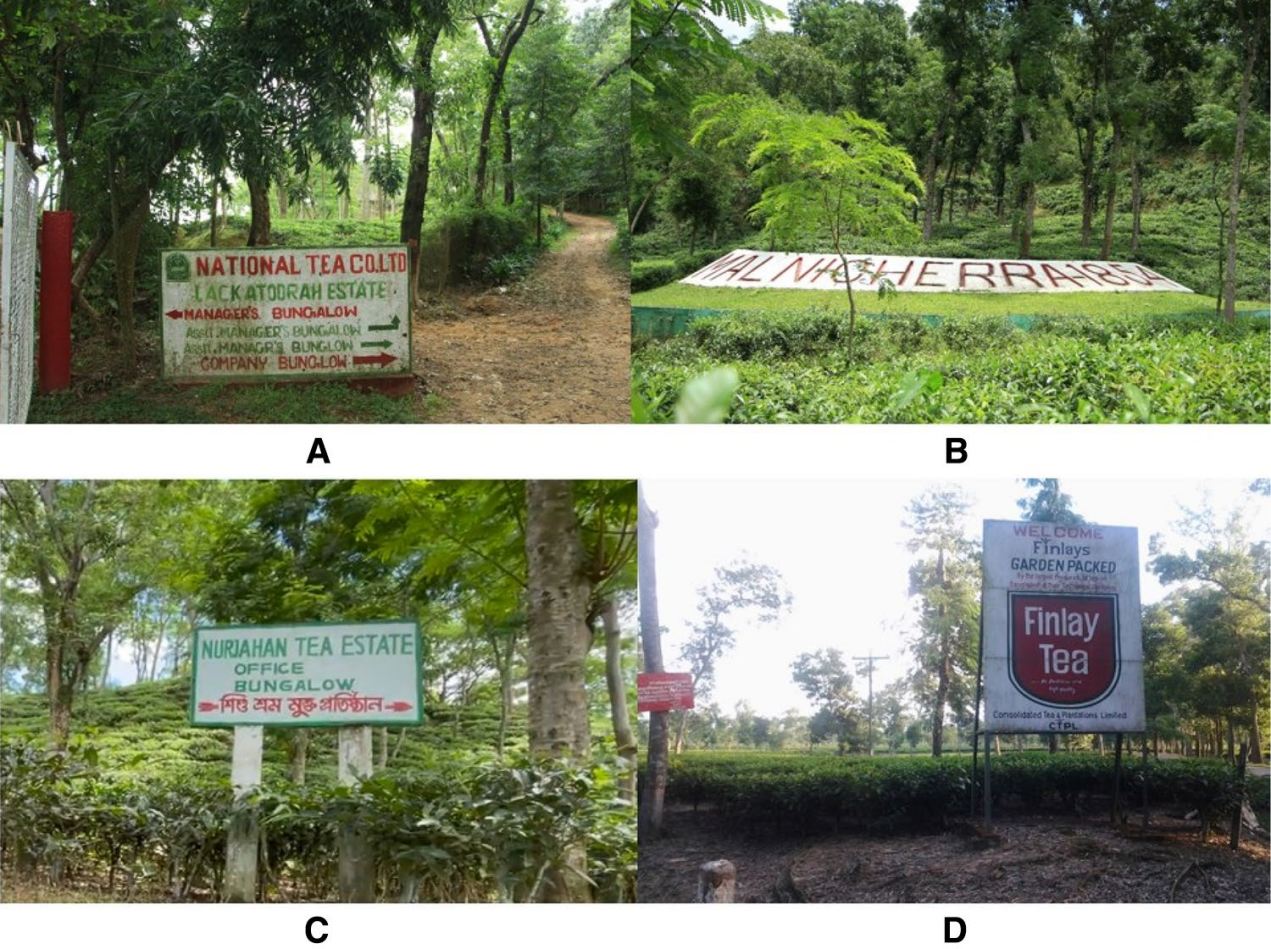
Research data collection places; (**A**) National Tea Company Ltd., (**B**) Malnicherra Tea Garden, (**C**) Nur Jahan Tea Garden, (**D**) Finlay Tea Estate.

**Figure 2:**
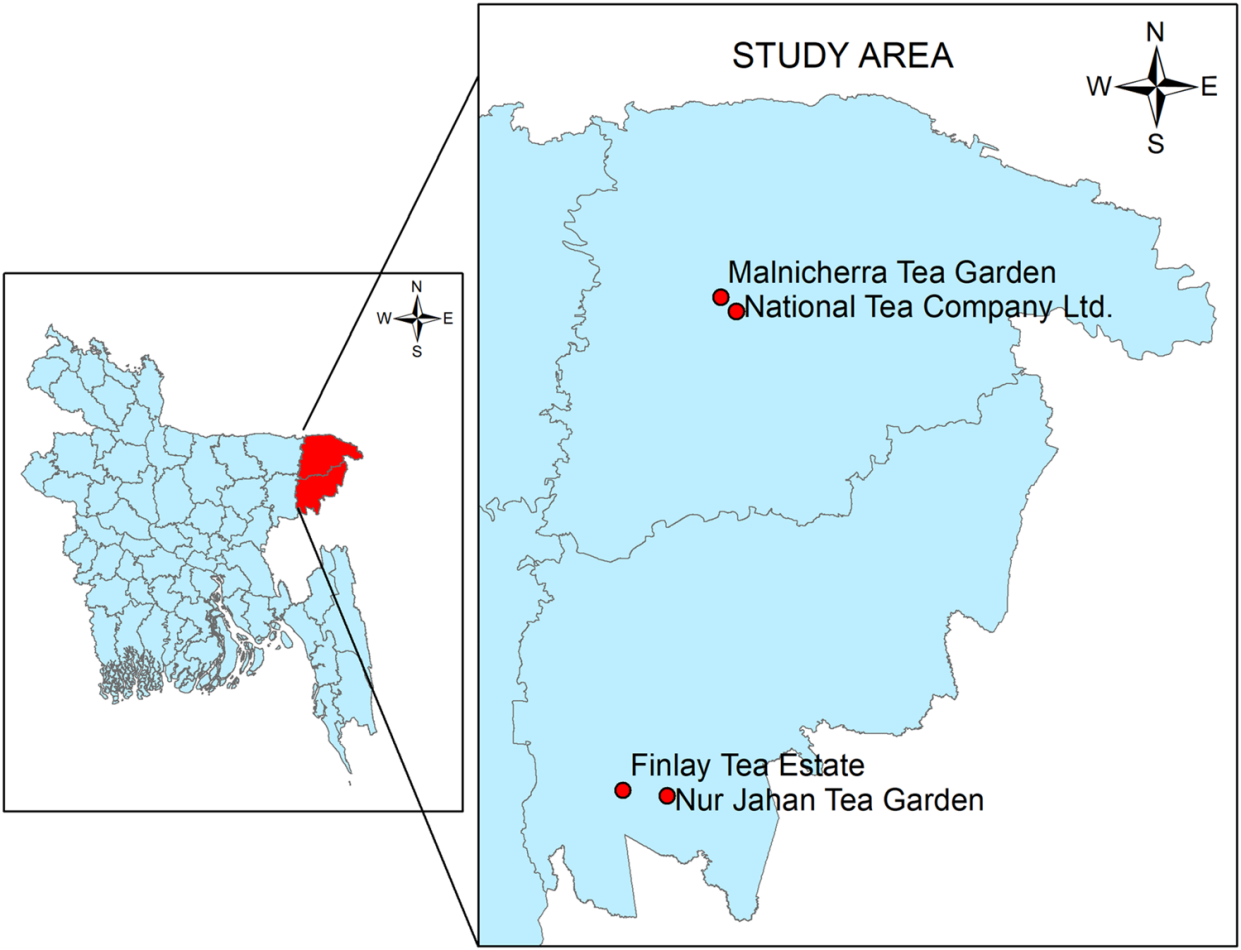
The geographical locations of four tea gardens studied in this research in Sylhet, Bangladesh.

Tea leaves were collected from four renowned tea gardens in the Sylhet district of Bangladesh as shown in Fig. 1. The geographical position of these four gardens is depicted in Fig. 2 so that the distance between them may be comprehended (**ArcGIS 10.8)**. The leaves were collected in June 2022 from two tea gardens, namely National Tea Company Ltd. (24°55′11.9″ north latitude and 91°52′25.7″ east longitude) in Lackatoorah, Sylhet, and Malnicherra Tea Garden (24°56′11.2″ north latitude and 91°52′01.2″ east longitude) on Airport road in Sylhet. Further, this research is extended to two more tea gardens, and leaves were collected during August 2022 from the gardens, namely Nur Jahan Tea Garden (24°17′50.5″ north latitude 91°48′05.6″ east longitude) and Finlay Tea Estate (24°19′12.0″ north latitude and 91°44′35.4″ east longitude), both in Sreemangal, Sylhet.

### Image acquisition and dataset building

**Figure 3:**
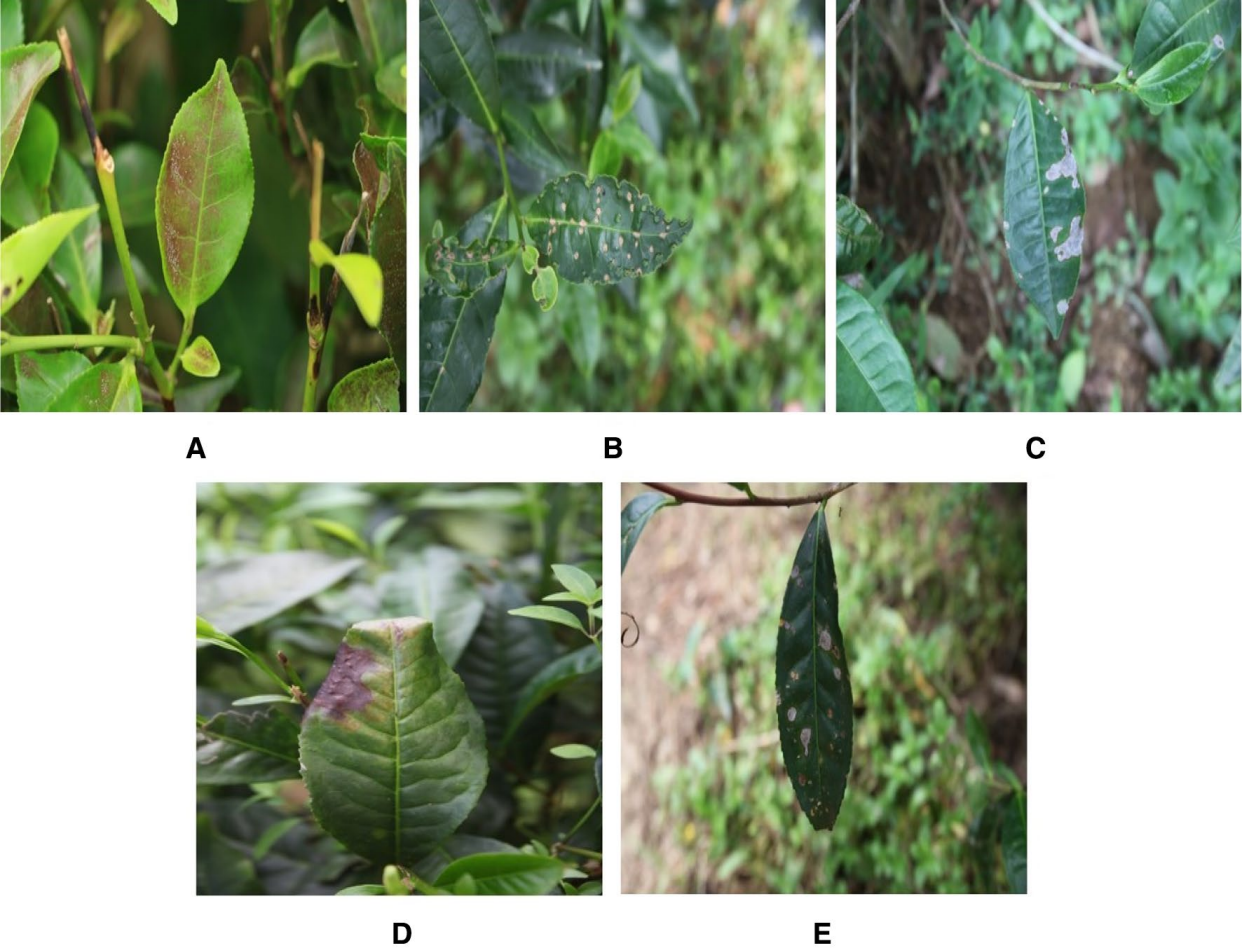
Images of tea leaf diseases: (**a**) red spider, (**b**) tea mosquito bug, (**c**) black rot, (**d**) brown blight, (**e**) leaf rust.

The experimental and field research methodologies utilized in this study were conducted in accordance with applicable rules and guidelines. During the study period, only images of diseased tea leaves were collected; no other collecting or sampling methods were used. The photos were taken in a natural environment using a Canon EOS 80D SLR camera with an image resolution of 6000 × 4000 pixels. The camera was positioned 0.4 m above the canopy of tea trees. From the images of diseased tea leaves captured in their natural surroundings, 4000 images of five types of tea leaves (infected with diseases) were chosen to generate a dataset for this study. Among these 4000 images, 800 images (each) of leaves infected by pests and diseases like red spiders, tea mosquito bugs, black rot, brown blight, and leaf rust. Figure 3 depicts images of these five tea leaf diseases taken from tea leaves. Initially, 800 images were randomly selected from 4000 images to evaluate the generalization of the detection model. The remaining 3200 images were randomly divided into a training set (2800) and a validation set (400). Since the image sizes in our dataset were not uniform, an initial normalization phase is done to standardize all photos to a 640 × 640 resolution image. To complete the manual labeling of the disease/infection, the image data annotation software 'Labeling' was used to create the outer rectangle of the diseased portions in all training set images using the 'labeling’ package in python. After the successful installation, image labelling (drawing the bounding box and labelling the class) is done for each image. After successfully labelling the image, the output is stored as a text file and a class file. To guarantee that the rectangle comprises as little of the backdrop as possible, images were labeled based on the smallest surrounding rectangle of the tea leaves. The diseased tea leaves were handled with care to prevent their mixing.

### Training the model

**Figure 4:**
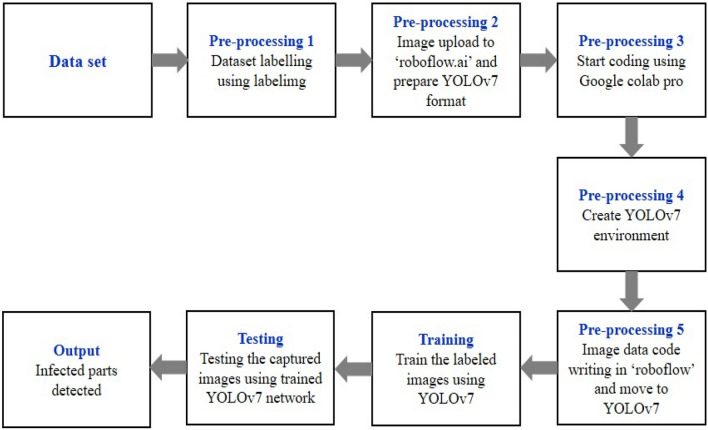
Block diagram of training and testing the proposed YOLOv7 model.

The YOLO package is installed by getting the 'YOLOv7' code from GitHub and cloning it. The newest version of 'YOLO v7' is supported by Torch and can be easily implemented with the help of 'Google colab'. This will generate a new folder on the system named 'YOLOv7'. This new folder will store the model's pre-trained weights and the special YOLO directory structure. A new subfolder is made in YOLOv7 once training is complete. To add the path of the location of the subfolder, the notation 'YOLOv7/run/training/experiment/weights/last.pt' is used. The size of the weight of the document will be changed according to the 'yaml' document that was used here. A block diagram of the testing and training framework of the proposed model is given in Fig. 4.

Following are the specifics of training the YOLOv7 model.

- *Photo size*: 640 × 640
- *Number of images in each batch*: 10
- *Feature extraction*: data.yaml
- *Developed Yolo*: YOLOv7s.yaml
- *Image*: measurement of an image's dimensions (in both height and width).
- *Batch*: batch size is the number of images fed in at once during an iteration.
- *epochs*: The amount of training repetitions or iterations.
- *Data*: the data structure, including the location of the training and validation data, the total number of classes, and the names of each class was characterized in this YAML file.
- *cfg*: To learn more about a model, you can look at its YAML configuration file in the 'model' folder. There are 4 distinct models available, each with a unique size range. The training file named "YOLOv7s.yaml" has been used.
- *Name*: There is a given model name.
- %cd yolov7/# change the name of the directory to ‘yolov7’ by using command
- !python train. py --img 640 --batch 10 --epochs 205 --data/content/data.yaml --cfg models/yolov7s.yaml --name TeaLeafDisease

Throughout the training process, data was gathered, the loss was analyzed, and the model weight was captured at each epoch with the help of the Tensorboard visualization tool. The following desktop computer specifications (Table 1) were used for training and testing using the PyTorch deep learning framework.

**Table 1:** Experimental conditions, hardware configurations and software packages.

Experimental environment	
Processor	Inter Core i7-7800X CPU
Operating system	Windows 10
Ram	32 GB
Graphics card	TUF Gaming GeForce GTX 1630 4 GB
Programming language	Python 3.8
Deep learning libraries	TensorFlow and PyTorch 1.8.2
Software	CUDA 11.4 + CUDNN 8.2 + OpenCV 4.5 and Visual Studio

### YOLOv7 architecture

YOLOv7 is derived from the YOLOv4, Scaled YOLOv4, and YOLO-R model architectures. The YOLOv7 model preprocessing strategy is combined with the YOLOv5 model preprocessing technique, and mosaic data augmentation is appropriate for identifying small objects. In terms of architecture, expanded ELAN (E-ELAN) is proposed as an extension of ELAN. The computational block of YOLOv7's backbone is known as E-ELAN. Expand, shuffle, and merge cardinality are applied to continuously enhance the network's capacity for learning without compromising the gradient route. Group convolution is utilized to increase the channel and cardinality of the computing block in the architecture of the computing block. Different sets of computational blocks are instructed to acquire various features. YOLOv7 also introduces compound model scaling for concatenation-based models. The method of compound scaling allows for the preservation of the model's starting attributes and, consequently, the best structure. Then the model concentrates on several trainable optimization modules and techniques known as "bag-of-freebies" (BoF)^27,36^. BoF is strategies that improve a model's performance without raising its training cost. YOLOv7 has implemented the following BoF approaches.

### Planned re-parameterized Convolution

Re-parameterization is a technique for enhancing a model following training. It lengthens the training duration but improves inference results. There are two methods of re-parameterization to complete models: model-level ensemble and module-level ensemble. Consequently, Module level re-parameterization has garnered significant interest in the scientific community. In this method, the process of model training is divided into various modules. The outputs are aggregated to produce the final model. YOLOv7 use gradient flow propagation channels to identify the model segments (modules) that require re-parameterization. The architecture's head component is based on the concept of multiple heads. Consequently, the lead head is accountable for the final categorization, whilst the auxiliary heads aid in the training procedure^21^.

### Coarse for auxiliary and fine for lead loss

**Figure 5:**
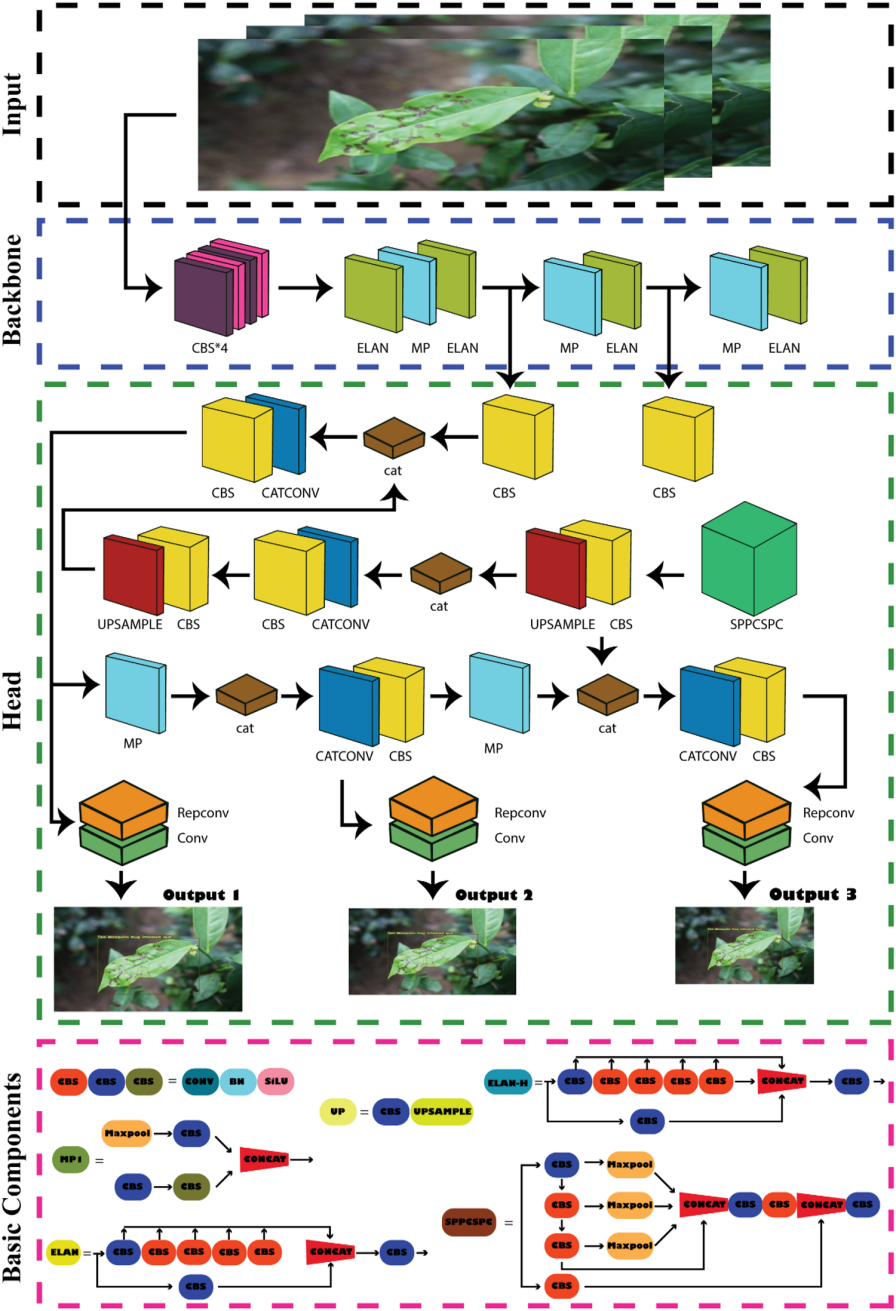
Network architecture diagram of YOLOv7. The whole architecture contains 4 general modules, namely, an input terminal, backbone, head, and prediction, along with 5 basic components: CBS, MP, ELAN, ELAN-H.

The projected model outputs are located at the top of YOLO. YOLOv7 is not confined to a single head, as it was inspired by deep supervision, a common training strategy for deep neural networks. It has several heads to accomplish anything it desires. The head responsible for the ultimate output is the lead head, whereas the head employed to support training in the middle layers is referred to as the auxiliary head. To improve the training of deep neural networks, a Label Assigner mechanism was designed that assigns soft labels based on network prediction outcomes and the ground truth. Traditional label assignment uses the ground truth directly to create hard labels based on preset criteria. Reliable soft labels, conversely, use calculation and optimization methods that consider both the ground truth and the quality and distribution of prediction output^27,36^. Figure 5 shows the overview of the network architecture diagram of YOLOv7.

### Steps of normalization

(a) The batch normalization layer is directly coupled to the convolution layer. This indicates that the batch's normalized mean and variance are added to the deviation and weight of the convolution layer during the inference step, (b) Using the addition and multiplication technique of knowledge acquisition in YOLO-R in combination with the convolution feature map, it can be standardized into vectors by pre-computation in the inference stage to be combined with the deviation and weight of the previous or subsequent convolution layer, and (c) finally, real-time object detection can considerably boost the detection accuracy without affecting the computational cost, so that the speed and precision in the range of 5–160 FPS surpass all known object detectors, enabling rapid response and accurate prediction of object detection^27,36^.

## Experimental results and analysis

### Image and label database

**Figure 6:**
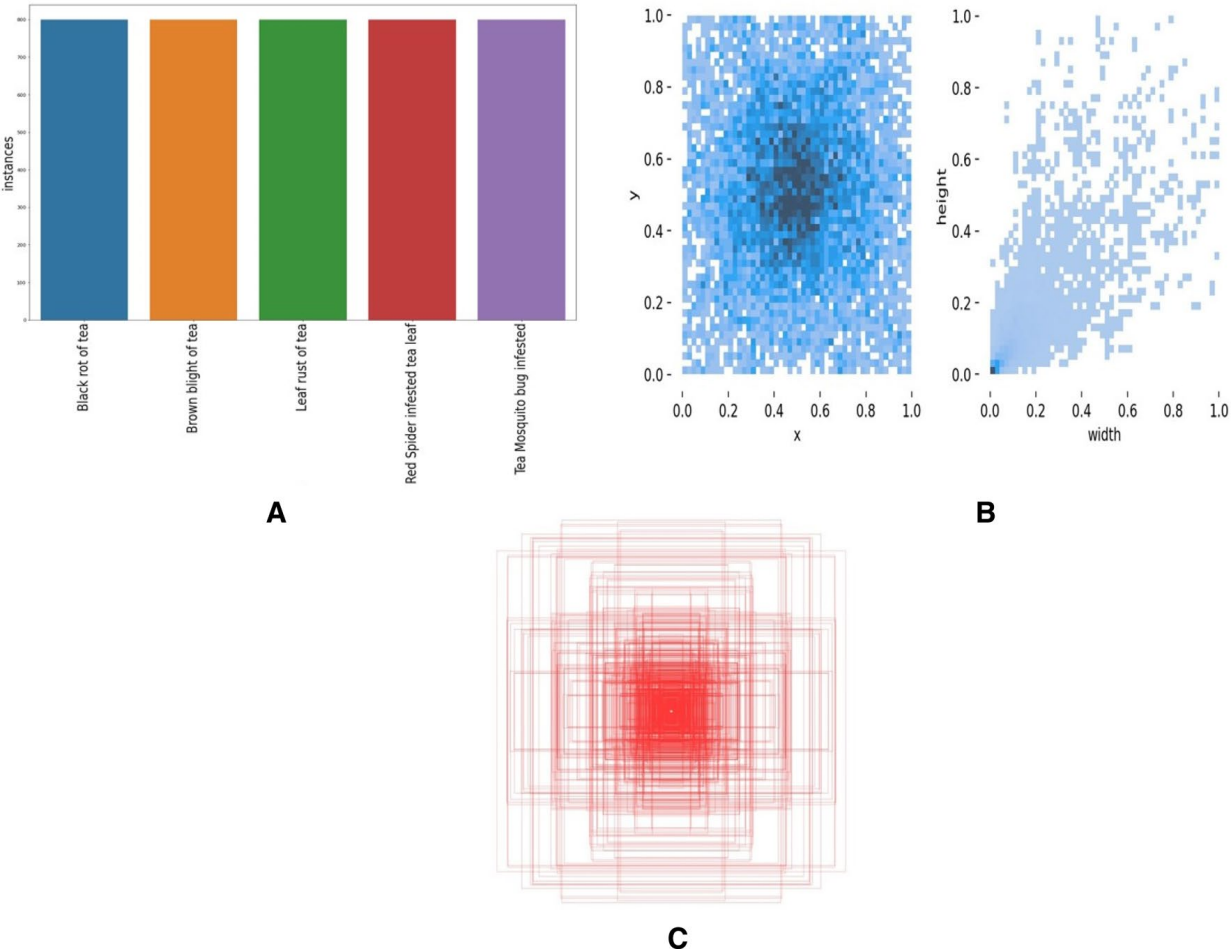
Labels and label distribution, (**a**) number and class of labels in the dataset, (**b**) location of the labels in the images of the dataset and the size of the labels in the dataset, (**c**) ground truth box.

The labeling tool was used to label the ground truth box of the images. The number and distribution of dataset tags were counted, and the result is shown in Fig. 6. This figure depicts a display of the augmented dataset's attributes. To improve the generalization of the trained model, the data augmentation enhances the information in the training dataset, maintains data diversity, and adjusts the distribution direction in the original images. The ordinate axis in Fig. 6a represents the quantity of labels, while the abscissa axis represents their names. The dataset contains sufficient amounts of samples of tea leaves that are infected and diseased. Figure 6b displays the tag distribution. The ordinate ‘y’ is the label center's abscissa ratio to the image height, and the abscissa ‘x’ is the label center's abscissa ratio to the picture width. The data is evenly and finely dispersed and focused in the middle of the image, as seen in the figure. The tea leaf dataset contains labels for ground-truth boxes (Fig. 6c). The size statistics of all image borders are shown in this figure. A clustering algorithm generates anchor boxes of varied sizes based on all ground-truth boxes in the dataset, ensuring that the initial anchor box size of the algorithm matches the intended size of the diseased tea leaves. In this case, the ground truth box refers to the boxes annotated around each instance of tea leaf disease in the training dataset. During the training, the YOLOv7 algorithm used these ground truth boxes to learn how to detect objects of similar classes in new images. The bulk of the boundary boxes in Fig. 6c is centred. The YOLOv7 algorithm uses anchor boxes to help locate objects, and placing these anchor boxes potentially results in the system finding things toward the image's center more frequently.

### Dataset training

**Figure 7:**
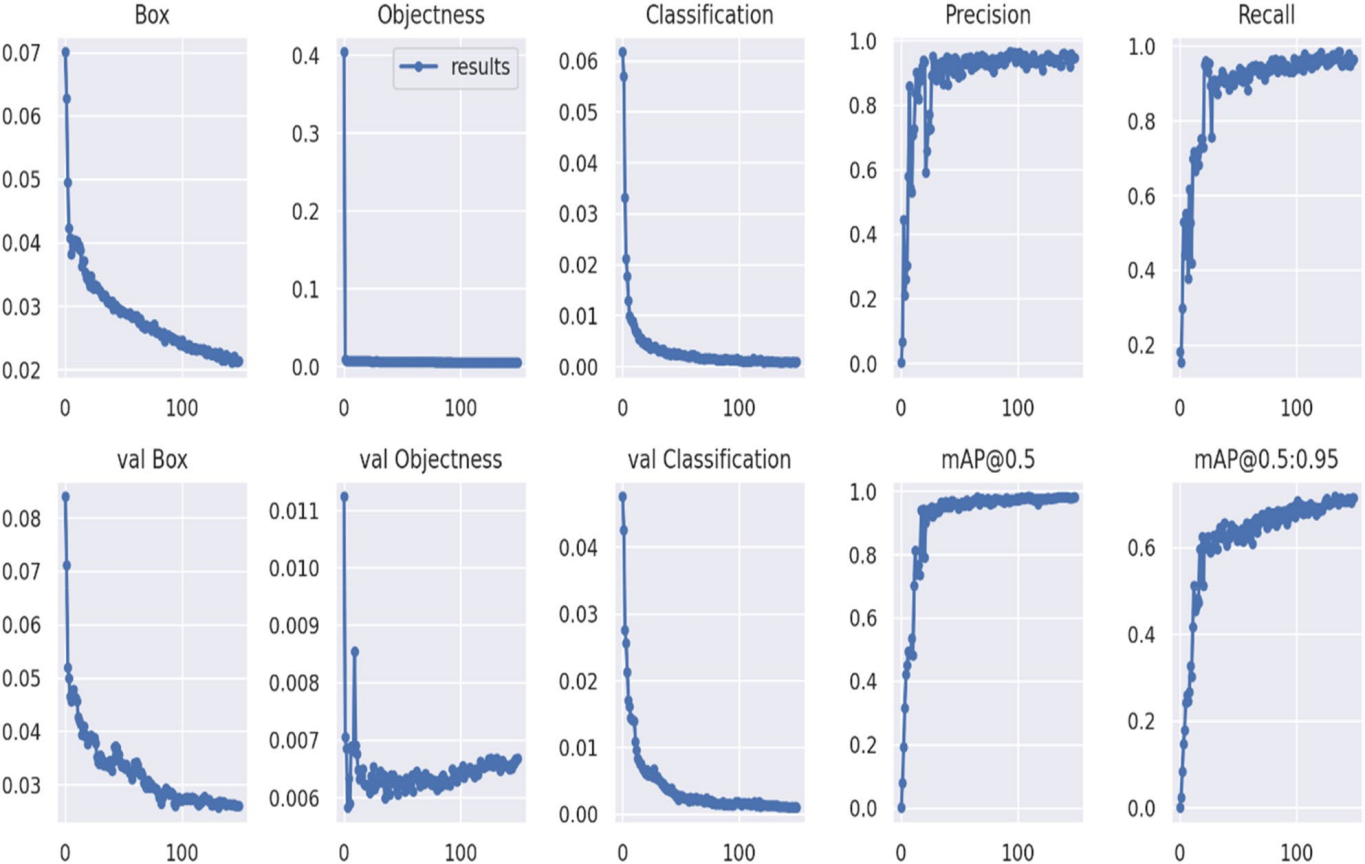
Visual analysis of model evaluation indicators (Precision, recall, and mAP@0.5 for the proposed YOLOv7) during training.

The original dataset and the YOLOv7 network were used to build the model for disease detection in tea leaves. The developed model's efficacy is shown in graphs, which show different metrics of the performance of training and validation sets. Three separate types of loss are depicted in Fig. 7**:** box loss, objectness loss, and categorization loss. The box loss assesses an algorithm's ability to precisely locate an object's center and estimate its bounding box. As a metric, "objectness" quantifies how likely an object can be found in a given area. High objectivity suggests that it is likely that an object lies inside the visible region of an image. Classification loss indicates the accuracy with which an algorithm can determine the proper class of an object. In the course of 0–100 iterations, the model's parameters vary considerably. When the number of iterations increased from 100 to 150, the model's performance was continuously optimized. The objectness loss is negligible, as the figure shows, YOLO v7 gives higher precision and recall values than K-Means.

### Performance measures

**Figure 8:**
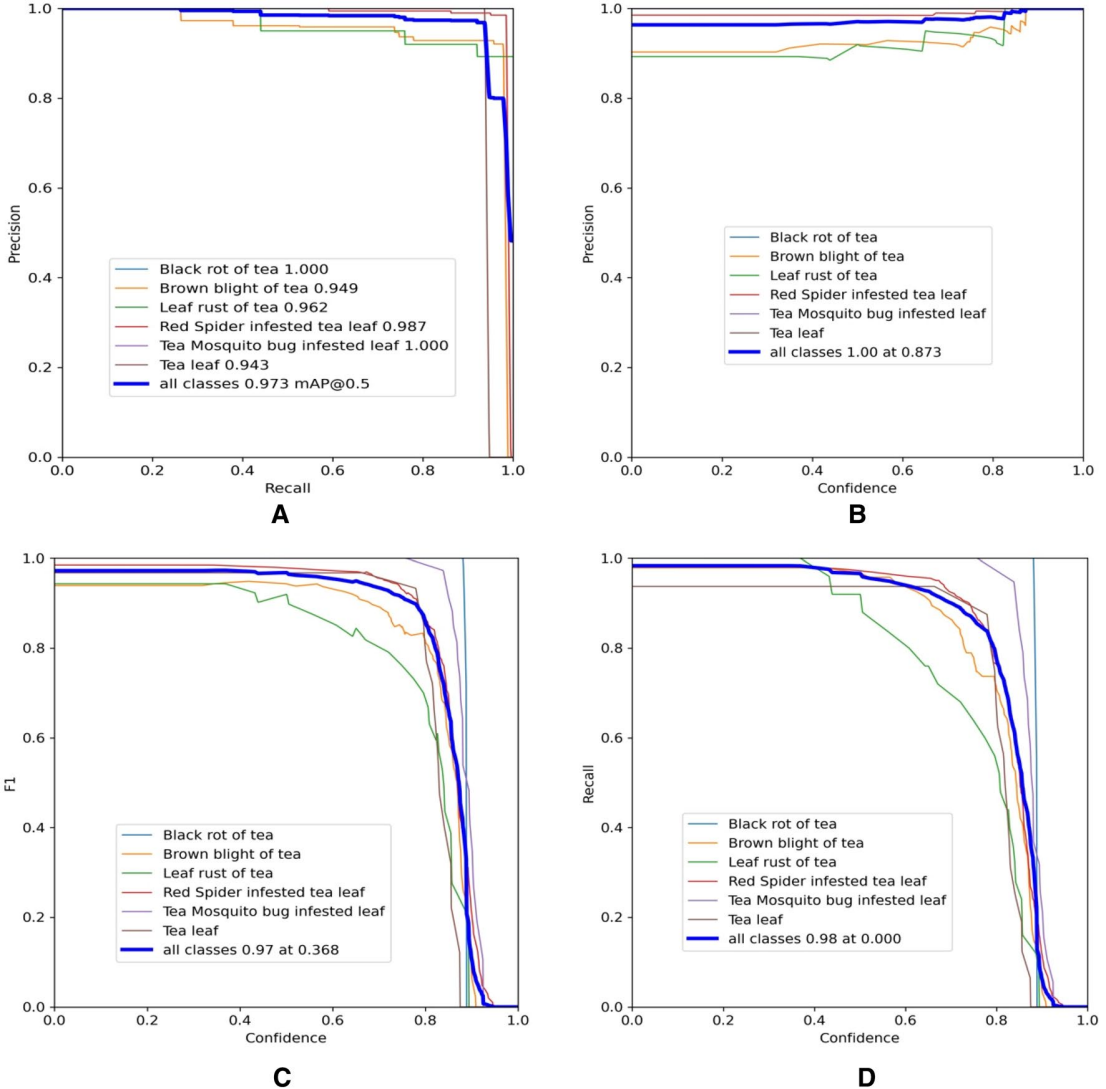
Operation results curve; (**a**) precision-recall curve, (**b**) precision-confidence curve, (**c**) F1-confidence curve, and (**d**) recall-confidence curve.

Precision and recall cannot be viewed as the sole determinants of the performance of a model because they could mislead regarding the model's performance^41^. Therefore, we employ additional curves to assess the performance of the model is computed as shown in Fig. 8. the precision-recall curve is depicted in Fig. 8a, and b illustrates the precision (P) versus confidence (C) graph, Fig. 8c depicts the F1 score at 97% with the confidence of 0.368, which advocates the balancing of P and R based on the tea leaf disease images dataset. Figure 8d depicts the recall (R) versus confidence (C) graph.

It was observed that as the recall grows, the rate of change in precision also increases. If the graph's curve is close to the upper right corner, it shows that as recall increases, the drop in precision is not easily visible, and the model's overall performance has increased. However, with a threshold of 0.5, the mAP for all classes is high and accurately models 97.3% of detections. This indicated in Fig. 8 that the algorithm could be relied upon to detect and classify objects of interest appropriately. However, in the start of the training and testing phases, the algorithm had challenges due to the lack of representative data, but it steadily converged as more training epochs were completed.

**Figure 9:**
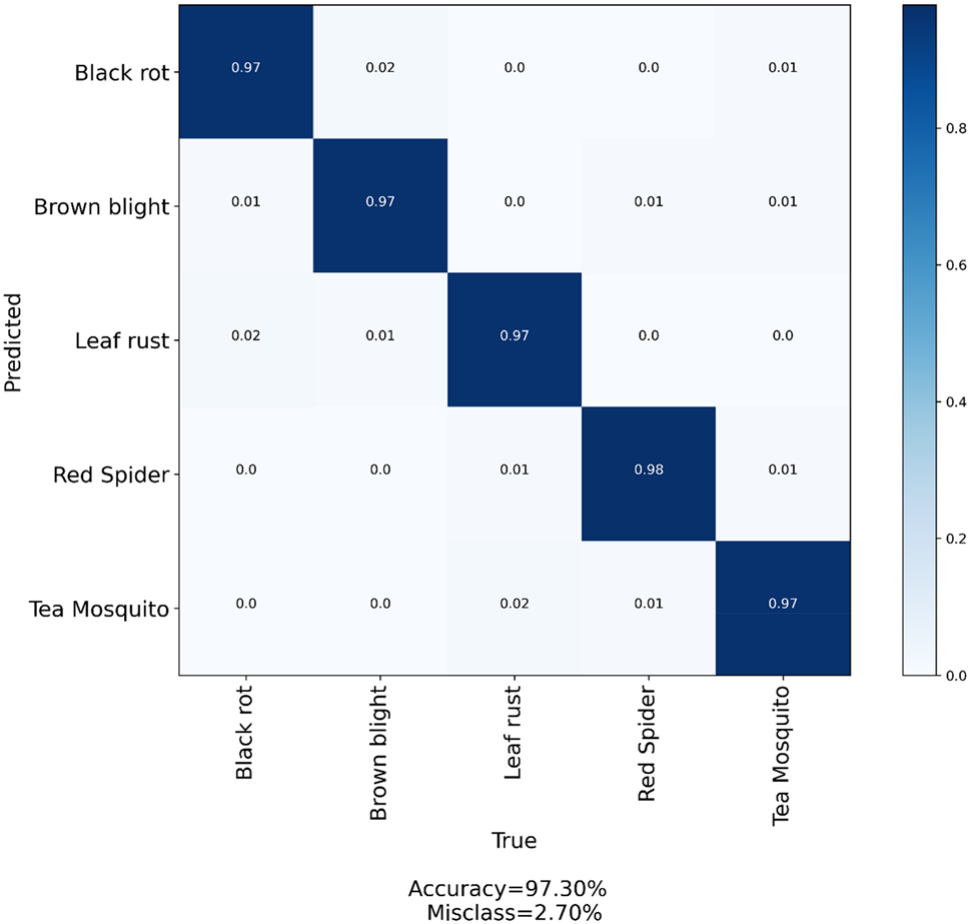
Confusion matrix diagram for the proposed YOLO-T model.

The confusion matrix in Fig. 9 contrasts the actual classification with the projected classification. It can illustrate where the model becomes confused while classifying or distinguishing between two classes. This is represented by a two-by-two matrix, with one axis representing the real or ground truth and the other representing the model's truth or the prediction. In a perfect scenario, 1.00 would span the diagonal from the matrix's upper left to lower right. The proper classification percentage for each type of diseased tea leaf according to the model appears to be as follows:

- *Black rot* 97%
- *Brown blight* 97%
- *Leaf rust* 97%
- *Red spider* 98%
- *Tea mosquito* 97%

In addition to showing the percentage of correctly classified algorithm output, it is also possible to see how many times the classification was wrong. Black rot, brown blight, and leaf rust were incorrectly categorized as leaf rust, black rot, and tea mosquito, respectively, 2% of the time, which caused the most confusion when classifying diseases.

### Comparison of models

The experimental results included four outcomes: true positive (TP), which refers to the accurate detection of individually marked diseased leaves; false positive (FP), which refers to an object that was incorrectly identified as a diseased tea leaf; true negative (TN) which refers to negative samples with a negative system prediction; and false negative (FN) which refers to diseased tea leaves that were overlooked. The YOLOv7 model of this present study is compared with YOLOv5 to confirm its accuracy and efficacy. Table 2 compares mAP, precision, recall, and training time between YOLOv7 and YOLOv5. Precision refers to the proportion of correctly recognized tea leaf diseases across all images. The recall rate is the proportion of accurately recognized diseased leaves in the dataset. The only challenge we encountered with the YOLOv7 model was that it required more time to train, whereas the YOLOv5 model required less. The other parameters (Table 2) are higher than YOLOv5. Prominent statistical Metrics are calculated using the following equations^35^.

1 $$Precision = TP/\left( {TP + FP} \right)$$

2 $$Recall = TP/\left( {TP + FN} \right)$$

3 $$AP = 1/11 * \sum r \in \left( {0,0.1,0.2, \ldots 1} \right) \;pinterp\left( r \right)$$

4 $$F1 - score = \left( 2 \right) /\user2{ }\left( {\left( {1/precision} \right) + \left( {1/recall} \right)} \right)$$

**Table 2:** Comparison of evaluation indicators between YOLOv5 and YOLOv7.

After 150 iterations of training	YOLOv5	YOLOv7
Precision (%)	95.4	96.7
Recall (%)	96.4	96.4
mAP (%)	97.7	98.2
F1-score	0.958	0.965
Detection accuracy (%)	96.1	97.3
Time to train	1 h 1 m 13 s	5 h 23 m 50 s

Based on the analysis and comparison of the prior series of experiments, it is feasible to conclude that the upgraded YOLOv7 algorithm presented in this study offers significant advantages in terms of detection accuracy. Despite a little drop in speed, this method can still meet the real-time requirements of practical tea leaf disease detection applications.

### Visualization and discussion

**Figure 10:**
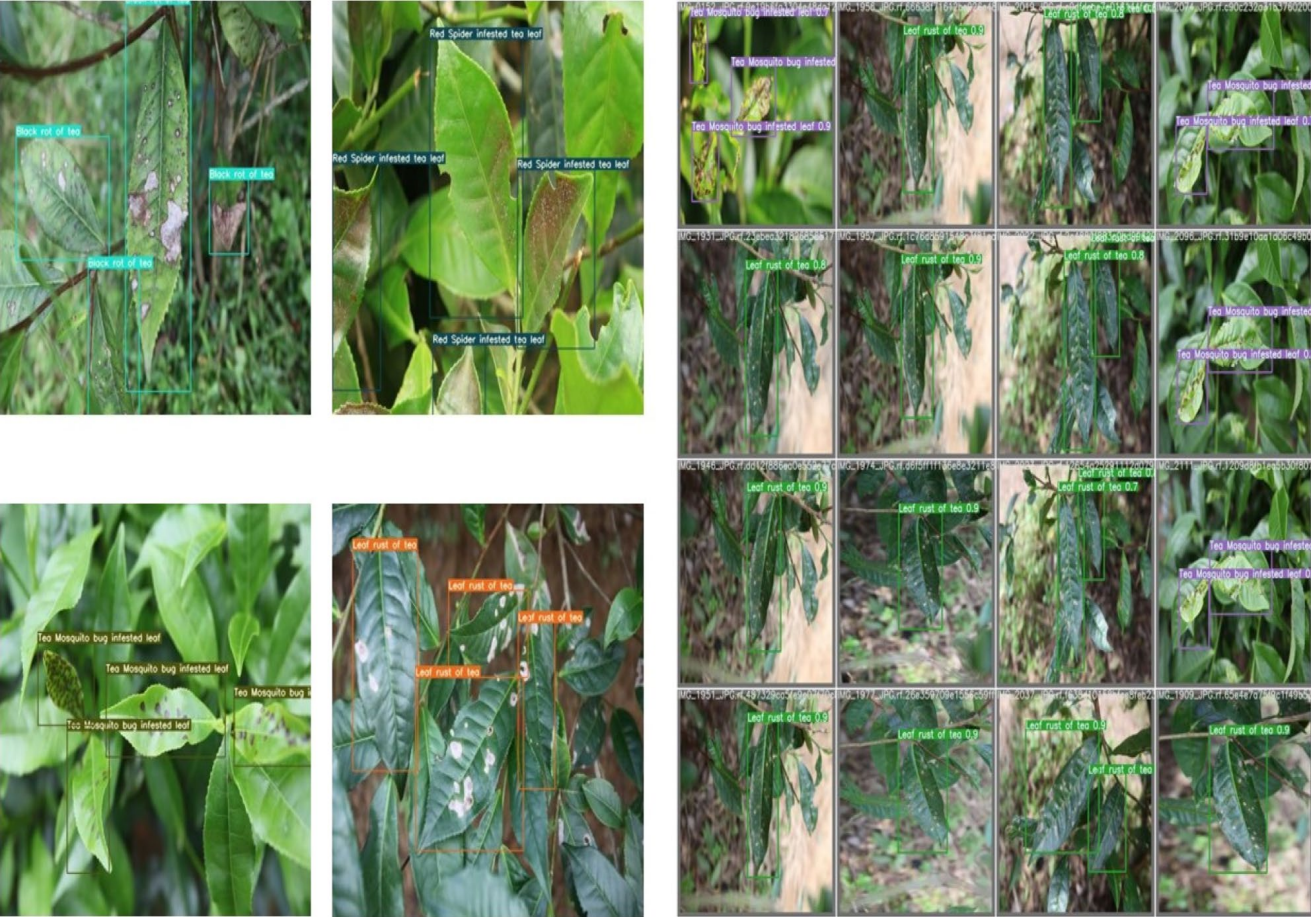
Some examples of tea leaf disease detection results using YOLOv7. The bounding boxes consist the images of diseased tea leaves.

The outcomes of the visualization of the identification of the five types of tea leaf diseases are shown in Fig. 10**.** This figure demonstrates that the proposed algorithm accurately detects and identifies diseased leaves by constructing a perfect bounding box. Deep Learning is gaining popularity among researchers for precision agriculture applications such as disease detection, weed control, fruit recognition, etc^45–47^. By identifying the diseased portion, farmers can employ more effective measures for disease control. The precision, recall, and average precision of this current YOLOv7 model are better than other object detection methods mentioned in the study of Hu et al.^48^. It employed a deep learning technique to identify and determine the severity of tea leaf blight disease^48^. His results were superior to those of other object detection algorithms; however, the performance of the current work is vastly superior to previous attempts.

In contrast, after reviewing the findings of the same study^48^, it was observed that the YOLOv3, faster R-CNN, and faster R-CNN + FPN appeared to require less training time^48^. The comparison of the outcomes of different tea leaf disease detection algorithms against the results obtained in this study is shown in Table 3. It can be observed that the detection accuracy and precision are much higher than the other similar studies, where researchers used different algorithms.

**Table 3:** Comparison of the outcomes of different tea leaf disease detection algorithms.

Model name	Diseases	Results	References
Deep CNN (LeNet)	1. Leaf blight 2. Blight disease 3. Red leaf spot 4. Red scab	Detection accuracy: 90.23% MCA: 90.16%	^49^
CNN	1. Algae leaf spot 2. Gray Blight 3. White Spot 4. Brown Blight 5. Red Scab 6. Bud Blight 7. Leaf blight	Detection accuracy: 94.45%	^50^
DNN	1. Gray Blight 2. Algal spot 3. Brown Blight 4. Helopeltis 5. Red Spot	Detection accuracy: 96.56% Precision: 96.63% Recall: 96.49% F1-score: 0.965	^51^
AX-RetinaNet	1. Algae leaf spot 2. Bud blight 3. White scab 4. Leaf blight	mAP: 93.83% Precision: 96.75% Recall: 94.00% F1-score: 0.954	^7^
Improved DCNN	1. Bud blight 2. Leaf blight 3. Red scab	Detection accuracy: 92.50%	^4^
CNN (LeafNet)	1. Bird's eye spot 2. Gray Blight 3. White Spot 4. Brown Blight 5. Red leaf spot 6. Algal leaf spot 7. Anthracnose	Detection accuracy: 90.23% MCA: 90.16%	^52^
Multi-objective image segmentation	1. Red Rust 2. Red Spider 3. Thrips 4. Helopeltis 5. Sunlight Scorching	Detection accuracy: 83% Precision: 77% Recall: 84% F1-score: 0.780	^53^
Improved YOLOv5	1. Tea cell eater (*Apolygus lucorum*) 2. Leaf blight	Detection accuracy: 91% Precision: 87.80% Recall: 85.27% mAP: 85.35% FPS: 51	^55^
YOLOv7	1. Red spider 2. Tea mosquito bug 3. Black rot 4. Brown blight 5. Leaf rust	Detection accuracy: 97.30% Precision: 96.70% Recall: 96.40% mAP: 98.2% F1-score: 0.965	Present study

Tea pests can be divided into three groups based on where they attack or infest, including root pests such as cockchafer grub, mealy root bug, and nematode; stem pests such as shot hole borer and red coffee borer; and leaf pests such as tea mosquito bug, flush-worm, looper caterpillar, leaf roller, thrips, and all mites. Diseases caused by the tea mosquito bug and the red spider are among Bangladesh's most significant threats to tea production, confirmed and concluded by other studies^54^.

Tea disease targets are often small, and the growing area's complex background easily impedes the procedure of their smart detection. In addition, several tea diseases are concentrated throughout the entire leaf surface, necessitating inferences from global data^55^. Regarding the leaf disease detection task, where accuracy was the essential factor, the proposed YOLO-T model is superior to other models. We discovered that some bounding boxes are too large for the disease area. The labelled name and prediction do not appear together in the image. This is because the name is configured to be excessively long, causing it to appear incomplete in the images. Correct annotation, labelling, and using a shorter, meaningful name resolved the issue. The bounding box must be drawn close to the required detection region of the object. This technique can assist the training algorithm in learning solely within the bounding box. Another benefit of this approach is its image resolution. The 640 × 640 image input size provides the maximum degree of precision^56^. The larger the size of the input image, the greater the amount of information it contains.

The only disadvantage we discovered while utilizing the YOLOv7 model was its lengthy training period. We compared our version (YOLO-T) to the most recent version (YOLOv5). A new study found that YOLOv7 required less training time than YOLOv5, which contradicts our findings^47^. This variance in training duration may be due to the utilization of graphics processing units (GPUs). Using a normal GPU can slow down YOLOv7's training time.

Except for the training time, the result of a recent study^55^ that also used the YOLOv5 version for the tea leaf disease was consistent with those of the present research. YOLOv5 employs a Focus structure that requires less Compute Unified Device Architecture (CUDA) memory, a reduced layer, and enhanced forward and backpropagation. It also uses a darknet backbone with a cross-stage partial network. On the other hand, E-ELAN in YOLOv7 utilizes expand, shuffle, and merge cardinality to obtain the capacity to constantly improve the network's learning ability without damaging the gradient route. According to a study, YOLOv7 has higher inference in speed and accuracy when compared with other algorithms such as YOLOR, PP-YOLOE, YOLOX, Scaled-YOLOv4, and YOLOv5 (r6.1)^57^. In several recent research, the detection accuracy and precision of the YOLOv7 algorithm have also been evaluated and reported^47,56,58–60^.

## Conclusions and future perspective

Identifying and detecting diseases is crucial to improving tea production during planting and harvesting. In the present era of high use of computation technology, an improved disease detection and identification system in the tea estates of a developing country like Bangladesh could have substantial potential for the country's economy, besides improving the farmers’ wealthy lifestyle. In this research study, the YOLOv7 model (YOLO-T) is used to detect and identify different types of tea leaf disease in tea gardens. The proposed model automatically detected five distinct types of tea leaf diseases and differentiated between healthy and diseased leaves. Overall classification accuracy is 97.30%, while recall and precision are 96.4% and 96.7%, respectively. The suggested model outperforms the most recent models reported in the discussion section regarding overall precision, accuracy, and recall. However, in this study, the performance of YOLOv7 is compared with the previous version, YOLOv5, and it was observed that YOLOv7 outperforms. Even though the outcomes are favorable, the proposed approach is limited by the duration of the training period. Future researchers can employ batch normalization for the next projects to accelerate the training process and improve precision. The expansion of the dataset is one of the focuses for future development. Future research should gather samples of damaged tea leaves from diverse varieties, fertility stages, and shooting angles in the field to compile a large dataset.

Moreover, image quality can be enhanced by employing more advanced labelling techniques. The model is compatible with Internet of Things (IoT) devices and applies to real-world applications. This framework can be slightly modified to account for additional crop diseases and adapted to other plants. The proposed algorithm can be implemented on a mobile application to facilitate farmers' access to assistance for their crops at any time. This research facilitates the early detection of numerous tea leaf diseases, which can contribute to their prompt detection. Subsequent studies may be focused on collecting temperature and humidity information, pathogenic spore information, soil information, and environmental parameters through multiple sensors, fuse multi-source data, and construct an early warning model of tea leaf diseases based on multi-data fusion to realize early warning when the disease does not occur.

## Data Availability

The datasets used and analyzed during the current study are available from the corresponding author upon request.
